# Beyond VAR2CSA: redefining the antigenic landscape of placental malaria vaccines

**DOI:** 10.12688/openresafrica.16523.1

**Published:** 2026-04-30

**Authors:** Lucy Mwai, Purity Gacheri Limbua, Jesse Gitaka, Bernard N. Kanoi

**Affiliations:** 1 1Centre for Malaria Elimination, Institute of Tropical Medicine, Mount Kenya University, Thika, Kenya; 2 Mount Kenya University School of Pure and Applied Sciences, Thika, Kenya

**Keywords:** VAR2CSA, placental malaria, placental malaria vaccines, Plasmodium falciparum, pregnancy-specific immunity, vaccine antigen mapping, multivalent vaccine

## Abstract

**Background:**

Placental malaria (PM) remains a leading driver of maternal and neonatal mortality, largely attributed to the sequestration of *Plasmodium falciparum*–infected red blood cells in the placenta. For decades, PM vaccine development has singularly focused on VAR2CSA, the immunodominant variant surface antigen. However, extreme polymorphism, limited cross-strain protection, and the structural complexity of VAR2CSA have hindered the development of a highly efficacious PM vaccine, necessitating a paradigm shift.

**Methods:**

This review examines existing literature to map potential vaccine antigens implicated in placental malaria ,and outlines a comprehensive vaccine development pipeline. It synthesizes emerging data on antigen expression profiles, immunogenicity, and mechanistic roles in placental pathogenesis. In addition, it highlights both key placental and blood-stage antigens expressed during pregnancy and reviews identification and testing strategies to guide rational PM vaccine design.

**Results:**

This Review maps the expanding antigenic landscape of PM beyond VAR2CSA, identifying non-canonical surface and exported proteins, including SURFINs, PHISTs, PfRON3, and *Pf*CSA-L, as critical targets that may circumvent current bottlenecks. The findings highlight the relevance of these antigens in placental malaria pathogenesis and their potential as alternative or complementary vaccine candidates.

**Conclusions:**

Furthermore, we propose an integrated framework for rational vaccine design that couples high-throughput antigen discovery and structural immunogen engineering with multivalent formulation strategies. By bridging the gap between molecular parasitology and translational immunology, this roadmap offers a strategic path toward a broadly protective placental malaria (PM) vaccine.

## 1. Introduction: The Persistence of Placental Malaria

Despite global strides in malaria elimination, placental malaria (PM) remains a recalcitrant and distinct pathology with devastating consequences for maternal and neonatal health. Caused by *Plasmodium falciparum,* PM is responsible for an estimated 10,000 maternal deaths and up to 200,000 infant deaths annually, primarily resulting from low birth weight (LBW), preterm delivery, and severe maternal anemia ( WHO, 2023; Zakama et al., 2020). Unlike systemic malaria, which affects the general population, PM is a parity-dependent syndrome; it disproportionately strikes primigravid women (first-time mothers) who lack the specific immunity developed during subsequent pregnancies. While current control interventions, such as intermittent preventive treatment in pregnancy (IPTp) and insecticide-treated nets, have reduced localized burdens, the emergence of drug-resistant parasites and vector insecticide resistance threatens to erode these gains. Consequently, the development of an efficacious vaccine remains a critical, yet unfulfilled, imperative for global health.

A defining hallmark of PM pathogenesis is the massive accumulation of infected red blood cells (iRBCs) within the placental intervillous space, a phenomenon driven by the specific adhesion of iRBCs to chondroitin sulfate A (CSA) on the syncytiotrophoblast surface ( Zakama et al., 2020). This sequestration allows the parasite to evade splenic clearance, triggering a localized inflammatory cascade characterized by the recruitment of monocytes and macrophages, placental insufficiency, and fetal growth restriction ( Mwai et al., 2024).

For over two decades, the quest for a PM vaccine has been synonymous with a single molecular target: VAR2CSA. A unique member of the *P. falciparum* erythrocyte membrane protein 1 ( *Pf*EMP1) family, VAR2CSA is the primary ligand mediating CSA binding ( Srivastava et al., 2011; Zakama et al., 2020). The prevailing dogma has been that blocking this interaction would suffice to prevent sequestration and its downstream pathology. Indeed, naturally acquired immunity in multigravid women is mediated by antibodies that target VAR2CSA and block adhesion, providing a compelling proof-of-concept for vaccine development ( J. Y. A. Doritchamou et al., 2023b; Srivastava et al., 2011).

However, translating this biological insight into a clinical product has proven exceptionally difficult. The sheer structural complexity of the VAR2CSA protein (~350 kDa), combined with its extensive antigenic polymorphism, has created significant bottlenecks. First-generation vaccines based on recombinant VAR2CSA fragments have shown safety and immunogenicity but limited cross-strain efficacy in clinical trials, highlighting the parasite’s capacity for immune evasion ( Mordmüller, 2019; Sirima, 2020).

### 1.1 Any vaccine interventions yet?

Malaria vaccine strategies have broadly targeted *Pf* at different stages of its complex life cycle, including pre-erythrocytic vaccines (PEVs), blood-stage vaccines (BSVs), and transmission-blocking vaccines (TBVs). PEVs aim to prevent liver infection following a mosquito and therefore target the sporozoites. The most notable approved PEVs given to children under 5 years are RTS,S/AS01 (Mosquirix) and R21/Matrix-M, both targeting the circumsporozoite antigen (CSP) ( WHO Weekly Epidemiological Record, 2023). The RTS,S/AS01 was the first malaria vaccine to be approved and deployed in 11 African sites in 2021 and has reported efficacy between 30–50% in children aged 5–36 months ( Agnandji et al., 2014). R21/Matrix-M received approval in 2023 and has shown over 70% efficacy in Phase II trials ( Datoo et al., 2021). However, these two vaccines have not been tested in pregnant women. Nevertheless, PEVs may help prevent malaria infection and thereby reduce the downstream occurrence of PM. One such vaccine is the radiation-attenuated *Pf*SPZ Vaccine ( *P. falciparum* NF54 sporozoites) by Sanaria Inc. that is under assessment for safety, immunogenicity and efficacy (phase 2 clinical trials - clinicaltrials.gov; NCT01441167) in women of childbearing potential and will later be tested in pregnant women ( Berry et al., 2025; Diawara et al., 2024; Jongo et al., 2023; Seder et al., 2013). On the other hand, BSVs aim to prevent symptomatic disease by blocking invasion of red blood cells by merozoites. Some of the promising targeted merozoite antigenic candidates include Erythrocyte Binding Antigen 175 (EBA175), *Pf* Reticulocyte-binding protein Homolog 5 ( *Pf*RH5), Apical Membrane Antigen 1 (AMA1), Merozoite Surface Protein 1 (MSP1) among others ( Abugri et al., 2022; Douglas et al., 2011; Kanoi et al., 2021; Salinas et al., 2019; Srinivasan et al., 2017; Thera et al., 2011; Weiss et al., 2015; Wipasa et al., 2002). Lastly, TBVs aim to interrupt parasite development in mosquitoes and include promising candidates like Pfs25, Pfs230, among others, which prevent fertilization, zygote formation and transmission to new hosts during mosquito blood meal ( Patel & Tolia, 2021).

**Figure 1.**
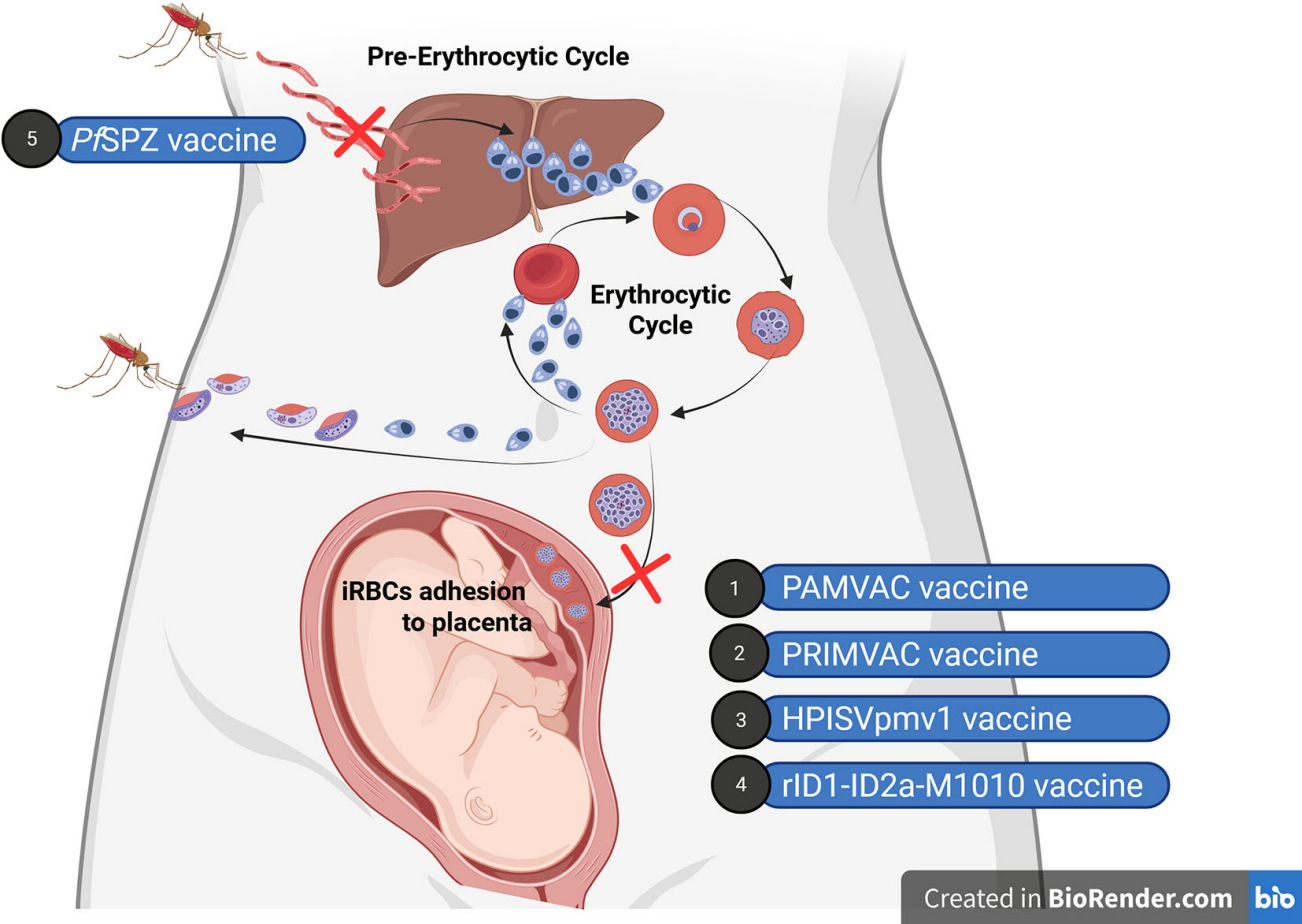
Current vaccines against placental malaria. Vaccine development for placental malaria (PM) has primarily targeted the Plasmodium falciparum VAR2CSA protein, responsible for infected red blood cells (iRBCs) adhesion to placental chondroitin sulfate A (CSA). First-generation candidates, (1) PAMVAC and (2) PRIMVAC, are VAR2CSA-based subunit vaccines currently in Phase I clinical trials. Structure-guided second-generation candidates, such as (3) HPISVpmv1, have shown enhanced immunogenicity in preclinical rat models, while (4) rID1–ID2a–M1010 demonstrates efficacy in Aotus nancymaae monkeys. Additionally, the (5) PfSPZ Vaccine, a radiation-attenuated sporozoite vaccine, is under Phase II evaluation to prevent infection upstream and potentially avert placental malaria. Created in BioRender.com

Efforts to develop vaccines specifically for PM have focused almost exclusively on VAR2CSA ( Fried & Duffy, 2015; Hviid, 2014). As outlined in Table 1 and Figure 1 there is no approved Placental Malaria Vaccines (PMVs) , although several promising candidates have been designed to block parasite binding to CSA ( Chêne et al., 2016; Gamain et al., 2021; Tuikue-Ndam & Deloron, 2015).

**Table 1.** Summarizing the key characteristics of placental malaria vaccines.

Vaccine name	Parasite antigen target	Design	Phase	Key findings	References
PfSPZ Vaccine -Non-placental malaria vaccine (non-PMV) specific	Attenuated *P. falciparum* sporozoites	Whole organism vaccine	• Testing in women of child bearing potential • Early safety trials in pregnant women underway	Well-tolerated, high efficacy in non-pregnant women	( Diawara et al., 2024; Seder et al., 2013)
PAMVAC - placental malaria vaccine	Based on ID1- DBL2x-ID2a VAR2CSA domain of FCR3 *P. falciparum* strain	• Recombinant subunit, *E. coli* expressed • Formulated with adjuvants Alhydrogel or GLA-based (GLA-SE or GLA-LSQ)	• Phase 1, 4th May 2016-9th March 2017 (4 dose Immunizations between 4th May 2016 to 30th August 2016) • 36 Healthy malaria naïve adult volunteers, • 2 centres; Germany and Benin	• Well tolerated/Safe and immunogenic; • Induced IgG with limited cross-reactivity • Highest Functional IgG response, with GLA-SE	( Mordmüller, 2019)
PRIMVAC - Priming malaria vaccine	Based on the DBL1x-DBL2x VAR2CA domain of 3D7 *P. falciparum* strain	• Recombinant antigen produced in Drosophila S2 cells • Formulated with Alhydrogel or GLA-based (GLA-SE or GLA-LSQ)	• Established in May 2016 • Phase Ia in malaria naïve population in France 18 to 35-year-old women • Phase Ib in nulligravid women from malaria endemic center in Burkina Faso • 3 dose immunizations	• Safe and immunogenic • Induced VAR2CSA-specific IgG; • Weak cross-inhibition of adhesion	( Sirima, 2020)
HPISVpmv1	Consists of Key CSA binding domains of VAR2CSA	Structure-guided recombinant design with improved yield and stability	Preclinical (Rodents, In vitro)	Preclinical studies showed that HPISVpmv1 is immunogenic and effective, and the adoption of a cocktail immunization strategy produced antibodies that worked against multiple strains.	( Ma et al., 2024)
ID1-ID2a M1010	Derived from ID1-ID2a construct of the VAR2CSA M1010 allele	Recombinant antigen vaccine	Preclinical (Aotus monkey model)	Boosted antibody titers but limited functional heterologous activity	( J. Doritchamou et al., 2023a)

The first PMV subunit vaccine is the Placental malaria vaccine (PAMVAC) candidate based on interdomain region 1 through the duffy binding like domain 2 to interdomains 2 (ID1- DBL2X-ID2a) fragment, a 73-kDa MW derivative of the VAR2CSA from the *P. falciparum* FCR3 clone, adjuvanted with Alhydrogel or glucopyranosyl lipid adjuvant in stable emulsion (GLA-SE) or in a liposomal formulation with QS21 (GLA-LSQ) and is expressed through the Drosophila melanogaster Schneider-2 (S2) cells ( Chêne et al., 2016; Pehrson et al., 2017). It was tested in healthy malaria naïve adult cohort Tubingen, Germany and malaria exposed nulligravidae in Cotonou, Benin. This was a randomized, double blind, phase 1 clinical trial where participants were immunized with 3 intramuscular injections of 20 μg (n = 9) or 50 μg (n = 27) every 4 weeks and follow up done for 6 months after the last immunization ( Mordmüller et al., 2019). The outcomes of the trial showed that PAMVAC was immunogenic in all volunteers with PAMVAC-specific antibody levels highest with PAMVAC-GLA-SE, and was safe and well tolerated. The purified antibodies from the plasma of the volunteers inhibited in vitro binding of VAR2CSA expressing *Pf* iRBCs to CSA functional assay ( Mordmüller et al., 2019).

The second VAR2CSA-based PMV vaccine candidate is the priming malaria vaccine (PRIMVAC) based on the DBL1X–DBL2X fragment, a 105-kDa derivative of the VAR2CSA from the 3D7 *P. falciparum* clone expressed as a recombinant antigen using Escherichia coli platform ( Chêne et al., 2016; Sirima, 2020). PRIMVAC adjuvanted with Alhydrogel® or GLA-SE underwent the first-in-human, randomized, double-blind, placebo-controlled, dose-escalation trials in France and Burkina Faso in May 2016, and yielded relatively safe, immunogenic and functional anti-VAR2CSA antibodies against the homologous VAR2CSA variant expressed by NF54-CSA infected red blood cells ( Cutts et al., 2020; P. E. Duffy & Patrick Gorres, 2020; Salamanca et al., 2019).

A novel, structure-guided vaccine candidate - the HPISVpmv1 - has also been designed ( Ma et al., 2024). It was developed from full-length VAR2CSA, retaining key CSA-binding domains, notably NTS, DBL1, DBL2, DBL4 - while eliminating immunodominant but highly polymorphic regions, such as DBL5 and DBL6 ( J. Y. A. Doritchamou et al., 2023b; Ma et al., 2021). Preclinical trials were done in rats, where a two-dose immunization regimen formulated with either CFA/IFA or AddaS03 adjuvant elicited antibody titers comparable or superior to the full-length VAR2CSA antigen and showed potent inhibition of iRBCs binding to CSA. Importantly, DBL4, which is excluded from PAMVAC and PRIMVAC, appears to contribute to improved functional activity ( Mordmüller, 2019; Salamanca et al., 2019).

Additionally, there is a recombinant antigen vaccine derived from the ID1-ID2a construct of the VAR2CSA M1010 variant, aimed at eliciting antibodies targeting the CSA-binding region of placental malaria parasites called rID1-ID2a-M1010 ( J. Doritchamou et al., 2023a). In the *Aotus nancymaae* model, the vaccine was administered with Alhydrogel adjuvant and compared to PMVs (PAMVAC, PRIMVAC), and a TBV negative control ( *Pf*s25). The immunization induced high antigen-specific IgG titers and modest surface reactivity to homologous VAR2CSA-expressing iRBCs, but exhibited limited cross-strain functional activity ( J. Doritchamou et al., 2023a).

### 1.2 Challenges in developing placental malaria vaccines for pregnant women

While VAR2CSA remains the principal vaccine candidate for placental malaria, its substantial allelic diversity presents a major hurdle to achieving broad vaccine efficacy. Structural and sequence variation among VAR2CSA alleles - including differences in domain number, CSA-binding affinity, and strain cross-reactivity - affects epitope presentation and immune recognition ( Renn et al., 2021). For instance, clinical trials with monovalent vaccine constructs namely PAMVAC and PRIMVAC have shown limited cross-strain protection, indicating that immunity acquired through vaccination may not be broadly neutralizing ( Mordmüller, 2019; Sirima, 2020). Moreover, atypical VAR2CSA variants with seven or more DBL domains, unlike the typical six, have been identified in field isolates and may obscure critical epitopes, facilitate immune evasion or worsen clinical malaria severity even in multigravid women with presumed immunity (P. E. Duffy & Patrick Gorres, 2020; Hviid, 2014).

Beyond the structural and sequence variation among VAR2CSA alleles, other emerging vaccine candidates, discussed earlier targeting blood-stage antigens like; EBA175, AMA1, MSP1 and pre-erythrocytic stages, have also been limited by high antigenic diversity, and poor immunogenicity ( Abugri et al., 2022; Weiss et al., 2015). Notably, AMA1 and MSP1 are polymorphic and elicit strain-specific immunity, while EBA antigens can be bypassed by alternative invasion pathways ( Abugri et al., 2022; Srinivasan et al., 2017; Thera et al., 2011; Weiss et al., 2015). TBV candidate antigens also face challenges related to scalability and validation of functional outcomes as they target vector stages.

Another complexity is the pregnancy-specific immunity. Despite prior exposure to malaria, primigravid women are highly susceptible to PM due to the unique expression of pregnancy-specific variant surface antigens like VAR2CSA ( Cutts et al., 2020). Protective immunity appears to be parity-dependent, with multigravidae expressing broader antibody responses ( Mwai et al., 2025).

Further, technical challenges during antigen expression, folding and epitope mapping limit vaccine development. The recombinant VAR2CSA is difficult to express due to its large multidomain structure. Expression in E. coli often yields misfolded proteins, while improved platforms like the wheat germ cell-free system (WGCFS) offer better fidelity can be costly ( Kanoi et al., 2021). Epitope dominance can compromise vaccine efficacy by directing immune response occurring toward non-protective regions, like what we see with subunit PMVs, failing to induce functional antibodies.

Additional challenges include transmission heterogeneity, host genetics, co-infections such as HIV, immune tolerance during pregnancy and ethical constraints further complicate vaccine performance and evaluation.

This review examines existing literature to map potential vaccine antigens that are implicated in PM, while also outlining a comprehensive vaccine development pipeline. It highlights both key placental and blood-stage antigens expressed during pregnancy and reviews identification and testing strategies to guide rational PM vaccine design.

## 2 Mapping potential vaccine antigens in placental malaria prevention

### 2.1 Introduction

The *Plasmodium falciparum* genome spans approximately 23 megabases and encodes more than 5,300 proteins across 14 chromosomes (M. F. Duffy et al., 2006; Dzikowski et al., 2006; Gardner et al., 2002). It is marked by a rich repertoire of antigen-encoding gene families, majority clustered within subtelomeric heterochromatic regions of chromosomes referred to as variable surface antigens (VSAs) ( Moya-Alvarez et al., 2014). The VSAs include the *var*, *rifin*, *stevor*, *surf*, *phist*, *pfmc-2tm*, and *hyp* families ( Su et al., 2019).

The *var* gene family comprises approximately 50–150 *var* genes that encode *PfEMP1* proteins which mediate cytoadherence to host endothelial receptors including CD36 (cluster determinant 36), ICAM1 (intercellular adhesion molecule 1), TSP (thromobospondin), CR1 (complement receptor 1), CSA (chondroitin sulphate A), α2-macroglobulin, complement 1q, E-selectins and P-selectins, endothelial protein C receptor (EPCR) and heparan sulfate, just to mention a few (M. F. Duffy et al., 2016; Dzikowski et al., 2006; Su et al., 2019). Such binding leads to severe manifestations like cerebral and placental malaria as well as activation of various host inflammatory responses (M. F. Duffy et al., 2006; Mwai et al., 2024). The regulation of *var* gene expression is monoallelic, with only one *var* gene expressed at a time ( Dzikowski et al., 2006; Flueck et al., 2009).

The r *ifin* gene family is the largest multigene family in *P. falciparum* with ~150–200 genes that encode the repetitive interspersed family proteins (RIFINs) ( Dzikowski et al., 2006). The *stevor* gene family comprises ~30–40 genes that encode the subtelomeric variable open reading frame proteins (STEVORs) ( Dzikowski et al., 2006; Sam-Yellowe et al., 2004). The *surf* gene family comprises about ~10 genes that encode surface-associated interspersed gene family proteins (SURFINs) ( Kanoi et al., 2020; Su et al., 2019). The *phist* gene family comprises >70 genes that encode *Plasmodium* helical interspersed subtelomeric proteins (PHISTs) ( Sargeant et al., 2006). The *pfmc-2tm* gene family comprises ~13 genes that encode the Pfmc-2TM proteins( Dzikowski et al., 2006; Sam-Yellowe et al., 2004). Together, the proteins are exported to the iRBC membrane or Maurer’s clefts (the specialized structures involved in protein trafficking to the RBC membrane), and sometimes on merozoite surface, with functional roles ranging from altering red blood cell rigidity to contributing to rosetting and evasion of host immunity ( Sam-Yellowe et al., 2004; Sargeant et al., 2006).

Structurally, *PfEMP1* proteins feature DBL and CIDR domains responsible for host receptor binding (M. F. Duffy et al., 2016). While RIFINs and STEVORs share structural motifs including a semi conserved N-terminal Plasmodium export element or host targeting signal (PEXEL) motif, transmembrane domain, and hypervariable C-terminal regions ( Dzikowski et al., 2006; Gardner et al., 2002). There are A-RIFINs that localize to the RBC surface, and B-RIFINs primarily intracellular but may be exposed on merozoites ( Kanoi et al., 2020). The STEVORs are slightly smaller (30–40 kDa versus 30–45 kDa) and are more conserved than RIFINs between parasite isolates ( Dzikowski et al., 2006). SURFINs on the other hand, are large proteins (200–300 kDa) with conserved C-terminal tryptophan-rich domains and variable N-terminal cysteine-rich domains ( Su et al., 2019). RIFINs, STEVORs, and SURFINs are expressed across multiple parasite stages, including merozoites, schizonts, gametocytes, and sporozoites, indicating roles in invasion and immune evasion ( Dzikowski et al., 2006). During schizogony and gametocytogenesis, they localize to Maurer’s clefts and are exported to the iRBC surface, influencing cytoadhesion and red blood cell rigidity ( Sam-Yellowe et al., 2004). PHISTs are α-helical and tryptophan-rich, classified into PHISTa, PHISTb, and PHISTc subfamilies based on conserved tryptophan residue patterns ( Sargeant et al., 2006; Templeton, 2009). These exported proteins likely interact with the red blood cell cytoskeleton, contributing to knob formation and red blood cell rigidity, with some implicated in *Pf*EMP1 anchoring during trophozoite and schizont stages ( Sargeant et al., 2006). The HYPs are largely unannotated but are predicted to be exported via the PEXEL motif and localized to the host cell; hence involved in host remodeling ( Sam-Yellowe et al., 2004). On the other hand, the *Pf*MC-2TM proteins have two transmembrane domains, localized to Maurer’s clefts ( Dzikowski et al., 2006; Gardner et al., 2002; Sam-Yellowe et al., 2004).

In addition to VSAs, *Pf* encodes several asexual blood-stage antigen families, including merozoite surface proteins (MSPs), red blood cell binding antigens (EBAs), reticulocyte binding-like homologs ( *Pf*Rh proteins), and cytoadherence-linked asexual gene products (CLAGs), that are essential for red blood cell invasion by mediating receptor-ligand interactions and activating invasion pathways ( Abugri et al., 2022; Salinas et al., 2019; Wipasa et al., 2002).

### 2.2 Potential vaccine antigens in placental malaria prevention

To strategically advance placental malaria vaccine development, it is critical to map the full antigenic landscape of *Pf*, particularly those implicated in malarial placental pathogenesis. The prior section has explored the parasite’s genome and key gene families, their structures, expression profiles, and immune relevance revealing a broader array of vaccine-worthy targets beyond VAR2CSA. The section below consolidates these insights by profiling leading antigen candidates as summarized in Table 2.

**Table 2.** Showing the leading and potential placental malaria vaccine antigen candidates using genomic, proteomic, functional, and immunological data to support rational vaccine design.

Gene ID	Antigen name/domain	Family	Parasite expression stage	Seroprevalence/immune data	Antigen function/localization/implication in PM	Vaccine potential/status	Reference(s)
PF3D7_1200600	VAR2CSA	PfEMP1	Trophozoite/Schizont	42–62% seroprevalence in malaria exposed pregnant women cohort in Kenya	Mediates CSA binding and placental sequestration of iRBCs The core CSA binding; DBLpam2, flanking ID1 and ID2/CIDRpam	PAMVAC, PRIMVAC, HPISVpmv1, ID1-ID2a M1010	( Fried & Duffy, 2015; Gangnard et al., 2019; Mordmüller, 2019; Mwai et al., 2025; Srivastava et al., 2011)
PF3D7_1001000 (formerly PF10_0013)	Chondroitin sulfate A ligand **(**CSA-L)	VAR2CSA-associated ligand	Surface late trophozoite/schizont	Anti-PfCSA-L IgG increased with gravidity, co-localizes with VAR2CSA	Highly conserved; Binds CSA and VAR2CSA Direct role in sequestration	Further research to assess anti-PfCSA-L for protective response	( Keitany et al., 2022; Rotich et al., 2025)
PF3D7_0936900 (formerly PFI1785w)	PHISTb - antigen	PHIST (Poly-Helical Interspersed Sub-Telomeric Exported antigens)	Ring, Trophozoite, and Schizont stages	Highly recognized by sera from malaria-exposed pregnant women; significantly higher in women vs men	Exported to the iRBC membrane; proposed to interact with cytoskeleton and PfEMP1 anchoring complex; localizes to Maurer’s clefts (iRBC trafficking organelles)	Conserved, immunogenic, and surface-exposed in placental parasites; potential complementary vaccine target to VAR2CSA	( Kamaliddin et al., 2017; Tuikue-Ndam & Deloron, 2015)
PF3D7_0424000 (formerly PFD1140w)	PHISTb antigen	PHIST (Poly-Helical Interspersed Sub-Telomeric Exported antigens)	Ring, trophozoite and schizont stages	Recognized by sera from pregnant women in endemic areas; co-immunoreactive with VAR2CSA	Exported to the iRBC membrane; thought to anchor and stabilize PfEMP1 complexes	Experimental Immunogenic, conserved, and surface-accessible; may complement VAR2CSA immunity	( Rotich et al., 2022; Tuikue Ndam et al., 2015; Tuikue-Ndam & Deloron, 2015)
PF3D7_0202400 (formerly PFB0115w)	*Plasmodium* Translation Enhancing Factor (PTEF), a translational regulator of VAR2CSA	Uncharacterized	Late trophozoite, schizont; peaks during schizogony	Detected in placental parasite transcriptomes. Immune reactivity data remains sparse	Potentially exported to iRBC surface; likely involved in cytoadherence or host cell remodeling, as inferred from gene clustering and expression profile	Exploratory. Given its expression profile and possible surface exposure, it is a candidate for future serological and functional immunogenicity studies	( Cutts et al., 2020; Keitany et al., 2022)
PF3D7_1201000	PHISTb Exported Antigen	PHIST (Poly Helical Interspersed Sub-Telomeric), subfamily b	Trophozoite	Not widely evaluated in pregnancy-specific cohorts;	Anchors PfEMP1 to host cell cytoskeleton; contributes to display of PfEMP1 variants like VAR2CSA at iRBC surface	Promising as a co-target with VAR2CSA; conserved, functionally essential, and potentially accessible to immune system	( Jonsdottir et al., 2021)
PF3D7_0424400	SURFIN 4.2	SURFIN	Trophozoite, Schizont, Merozoite	62% seroprevalence in malaria exposed pregnant women cohort in Kenya	Surface antigen, co-localizes with PfEMP1 on iRBC membranes; may contribute to sequestration or immune evasion mechanisms	High seroprevalence and differential expression in multigravida women suggest vaccine potential	( Mwai et al., 2025)
PF3D7_1301800	SURFIN 13.1	SURFIN	Trophozoite, Schizont stages	30% seroprevalence in malaria exposed pregnant women cohort in Kenya	Understudied antigen with hypothesized role in red blood cell cytoadhesion/sequestration in placenta	Lower seroprevalence but consistent immune recognition. Differentially expressed in multigravida women-vaccine potential	( Ito et al., 2021; Mwai et al., 2025)
PF3D7_1252100	PfRON3	RON	Merozoite stage (rhoptry neck)	69% seroprevalence in malaria exposed pregnant women cohort in Kenya	Forms a complex with PfRAMA to mediate tight junction formation during red blood cell invasion.	Strong immunogenicity; A promising blood-stage vaccine candidate. Rhoptry localization may elicit functional antibodies	( Ito et al., 2021; Mwai et al., 2025)
Monoclonal Antibody PDB: 7Z12 (VAR2CSA target)	PAM1.4 (targets conformational epitope in ID1, DBL2, ID2, DBL4)	Human monoclonal IgG to PfEMP1 (VAR2CSA)		Broad cross-reactivity to multiple VAR2CSA variants; identified in immune pregnant women	Binds conserved conformational epitope distant from CSA-binding site; does not neutralize adhesion but may mediate Fc-effector functions like ADCC and opsonization	Tool for epitope mapping; informs full-length VAR2CSA vaccine design; not a direct neutralizing vaccine candidate	( J. Y. A. Doritchamou et al., 2023b; Gangnard et al., 2019; Ma et al., 2021; Raghavan et al., 2022; Srivastava et al., 2011)

**2.2.1 VAR2CSA (PF3D7_1200600)**

VAR2CSA is a member of the PfEMP1 family and is expressed during the trophozoite and schizont stages. It mediates CSA binding and placental sequestration of iRBCs. Structurally, VAR2CSA comprises six Duffy binding-like (DBL) domains interspersed with interdomain (ID) regions, forming a large ectodomain capable of CSA recognition. In a recent study, the seroprevalence ranged between 42–62% in malaria-exposed pregnant women cohort in Kenya ( Mwai et al., 2025). Multiple vaccine constructs have been developed, including truncated fragments such as ID1-ID2a (used in PAMVAC and PRIMVAC), full-length variants like HPISVpmv1 or structurally guided constructs ( Ma et al., 2024; Mordmüller, 2019; Sirima, 2020). Despite high allelic diversity of VAR2CSA, and limited cross-strain antibody reactivity, VAR2CSA remains a central antigen due to its functional relevance and role in the pathogenesis of PM.

**2.2.2 PfCSA-L (PF3D7_1001000, formerly PF10_0013)**

PfCSA-L is a VAR2CSA-associated ligand expressed on the iRBC surface during the late trophozoite and schizont stages. It colocalizes with VAR2CSA on knob complexes and binds CSA directly. In a recent study, a statistically significant increase in anti- *Pf*CSA-L IgG levels across gestation suggests antibody maturation likely driven by placental parasite exposure ( Rotich et al., 2025). Genetic analyses of field isolates confirmed high conservation at both nucleotide and protein levels, and B-cell epitope prediction pinpointed immunogenic motifs within these conserved domains, reinforcing the candidacy of *Pf*CSA-L and its epitopes for further vaccine development ( Keitany et al., 2022; Rotich et al., 2025).

**2.2.3 PF3D7_0936900 (PFI1785w)**

The invariant gene PFI1785w encodes a PHISTb-type exported protein expressed across the ring, trophozoite, and schizont stages of the *Plasmodium falciparum* asexual cycle and has been identified as significantly overexpressed in placental malaria field isolates ( Kamaliddin et al., 2017). Initially discovered through transcriptomic and later confirmed via proteomic mass spectrometry approaches, PFI1785w is predicted to localize at the host red blood cell periphery, particularly within Maurer’s clefts ( Keitany et al., 2022). It likely contributes to the stabilization of cytoadherent complexes, potentially through interactions with the acidic terminal segment (ATS) of VAR2CSA. Its highly conserved sequence across global field isolates, coupled with strong serological reactivity in malaria-exposed women, underscores its immunological relevance and supports its candidacy as a conserved co-antigen in placental malaria vaccine design ( Kamaliddin et al., 2017).

**2.2.4 PF3D7_0424000 (PFD1140w)**

PF3D7_0424000, another member of the PHIST protein family, shares similar features with PFI1785w, being expressed throughout intraerythrocytic development and upregulated in PM-associated parasites ( Keitany et al., 2022). Like other PHISTb proteins, it is exported into the iRBC cytoplasm and localizes to the host cell membrane, where it is thought to assist in the -anchoring and stabilization of PfEMP1 - including VAR2CSA, via direct interaction with its intracellular ATS domain. Antibodies from malaria-exposed pregnant women show strong recognition of PF3D7_0424000, with notable co-immunoreactivity alongside VAR2CSA. These findings suggest it may enhance VAR2CSA surface presentation or act synergistically in parasite sequestration, making it relevant for multivalent placental malaria vaccine constructs ( Keitany et al., 2022; Rotich et al., 2022).

**2.2.5 PF3D7_0202400 (formerly PFB0115w)**

PF3D7_0202400 is an invariant gene that is upregulated in placental malaria parasites. It encodes a *Plasmodium* Translation Enhancing Factor (PTEF), a novel antigen hypothesized as a translational regulator of VAR2CSA expression ( Keitany et al., 2022). It is upregulated during the late trophozoite and schizont stages in placenta-infecting parasite isolates. Though immunological data remain sparse, its clustering with adhesion-related genes and possible surface localization suggest a cytoadherence-related function. As a novel candidate, it presents an exploratory target for serological profiling and functional validation in PM immunity.

**2.2.6 PF3D7_1201000**

This is another PHISTb subfamily protein expressed during the trophozoite stage and exported to the iRBC cytoplasm and has been shown to interact with RhopH components ( Jonsdottir et al., 2021). It interacts with the host membrane skeleton and facilitates PfEMP1 trafficking, thereby promoting display of PfEMP1 on the iRBC surface. Although its function is not yet clearly defined and it has not yet been evaluated in pregnancy cohorts, this protein has been identified as functionally essential in parasite survival and is potentially immunogenic.

**2.2.7 SURFIN 4.2 (PF3D7_0424400)**

SURFIN 4.2 belongs to the SURFIN family of antigens that are typically expressed on the surface of iRBCs and merozoites. This antigen is expressed predominantly during the trophozoite and schizont stages. In a recent immunoscreening study, it showed a seroprevalence of 62% in a cohort of pregnant women, indicating significant immune recognition in naturally exposed women ( Mwai et al., 2025). Functionally, SURFIN 4.2 co-localizes with PfEMP1 on the iRBC membrane and may participate in immune evasion or contribute to placental sequestration by mediating adhesion mechanisms. Despite being under-characterized, its expression profile and surface accessibility indicate it is a promising candidate for inclusion in multicomponent PM vaccines.

**2.2.8 SURFIN 13.1 (PF3D7_1301800)**

SURFIN 13.1 is another member of the SURFIN antigen family, expressed mainly during the trophozoite and schizont stages and is predicted to localize on the iRBC membrane. In a recent immunoscreening study, SURFIN 13.1 showed 30% seroprevalence, suggesting moderate but consistent immune exposure in naturally exposed pregnant women ( Mwai et al., 2025). Although its exact function remains to be elucidated, SURFIN 13.1 is hypothesized to play a role in cytoadhesion and cellular interactions due to predicted structural motifs ( Kanoi et al., 2020; Mwai et al., 2025).

**2.2.9 *Pf* Rhoptry Neck Protein 3 ( *PfRON3*) (PF3D7_1252100)**

*PfRON3* is a rhoptry neck protein expressed during the schizont and merozoite stages and plays a key role in red blood cell invasion. It forms a novel complex with *Pf* Rhoptry-Associated Membrane Antigen ( *Pf*RAMA), with their interaction critical for rhoptry neck biogenesis and the proper trafficking of *Pf*RON3 into rhoptries ( Ito et al., 2021). A knockdown of *Pf*RAMA disrupts *PfRON3* localization, indicating functional interdependence ( Ito et al., 2023; Low et al., 2019). Unlike Rhoptry Neck Proteins **(**RONs) - *Pf*RON2 and *Pf*RON4, which form part of the canonical AMA1-RON complex, *Pf*RON3 operates via a distinct invasion mechanism, further distinguishing its role in the early steps of merozoite entry ( Ito et al., 2021; Thera et al., 2011). *Pf*RON3 is also immunologically relevant having shown high seroprevalence (69%) among naturally exposed pregnant women and eliciting significantly stronger antibody responses in multigravidae compared to primigravidae ( Mwai et al., 2025). This gravidity-associated immune profile suggests it is a target of naturally acquired immunity and may be a promising candidate for parity-independent vaccine development.

**2.3.0 PAM1.4 (Protein Database Identifier: PDB - 7Z12)**

PAM1.4 is a human monoclonal antibody derived from immortalized B cells of malaria-exposed pregnant women that exhibits broad cross-reactivity to diverse *Pf* VAR2CSA variants ( J. Y. A. Doritchamou et al., 2023b; Ma et al., 2021; Raghavan et al., 2022). High-resolution cryo-electron microscopy and epitope mapping, have shown PAM1.4 binds a conformational epitope formed by multiple conserved regions in the VAR2CSA core structure; ID1, DBL2, ID2, and DBL4, but does not bind near the CSA binding site; DBL2x, 1D1/ID2, and also DBL3x, lacking CSA-inhibitory activity ( Gangnard et al., 2019; Raghavan et al., 2022; Srivastava et al., 2011). Despite not blocking CSA binding, its broad reactivity makes it an important tool for benchmarking immune responses and understanding the structural immunogenicity of VAR2CSA. For instance, its binding requires intact multi-domain constructs, reinforcing the value of full-length VAR2CSA antigens in vaccine development. Protective immunity against VAR2CSA thus may not depend solely on blocking parasite adhesion, but also on Fc-mediated pathways, such as opsonization and antibody-dependent cellular cytotoxicity (ADCC) to iRBCs ( J. Y. A. Doritchamou et al., 2023b; Raghavan et al., 2022).

### 2.3 Rational vaccine development strategies: A future perspective

The process of developing an effective placental malaria vaccine begins with antigen discovery. Transcriptomic and proteomic tools can be used to profile placental *Pf* isolates to identify antigen targets either overexpressed or functionally implicated in PM pathogenesis. Our team and others have used high-throughput serological platforms such as AlphaScreen and Luminex, and monoclonal antibody isolation, to prioritize antigens based on surface accessibility, conservation, and gravidity-dependent immune profiles ( Mwai et al., 2025; Rotich et al., 2025).

Another critical step is structure-guided immunogen design, which ensures prioritized antigens preserve functional conformational epitopes. Tools such as cryo-EM and nsEMPEM allow high-resolution mapping of antibody-binding domains. For example, the HPISVpmv1 vaccine construct was designed using this approach to include full-length ectodomains from multiple VAR2CSA alleles while excluding hypervariable domains DBL5–DBL6, improving immunogenic focus and expression yield ( Ma et al., 2024).

Following design, antigen production requires systems that ensure correct folding and post-translational modifications. The HPISVpmv1 vaccine was produced in mammalian cells, while WGCFS was used for PfCSA-L, SURFIN4.2, SURFIN13.1, and PfRON3, offering superior antigenic integrity over *E. coli* platform ( Kanoi et al., 2021; Mwai et al., 2025).

Functional assays are then needed to assess biological relevance. For instance, CSA-binding inhibition assays evaluate antibodies targeting placental-binding antigens (e.g., VAR2CSA, PfCSA-L), while growth inhibition assays (GIAs) test the ability of antibodies to block red blood cell invasion by merozoite antigens like AMA1 or *Pf*RON3 ( Srinivasan et al., 2017). Fc-effector function assays, including opsonization and antibody-dependent cellular cytotoxicity (ADCC), are also vital.

Formulation strategies must address antigenic diversity. Multivalent vaccines (e.g., VAR2CSA cocktails) and synergistic combinations (e.g., VAR2CSA + PfCSA-L or AMA1-RON2L) show promise but require empirical validation to avoid antagonism ( Salinas et al., 2019). Adjuvant selection and dosing schedules must be optimized for gravidity-specific responses ( J. Y. A. Doritchamou et al., 2021; Gamain et al., 2021).

Vaccine platforms under evaluation include subunit vaccines (PRIMVAC, PAMVAC), passive monoclonal antibodies (PAM1.4), and live-attenuated candidates (PfSPZ) ( Ma et al., 2024; Mordmüller, 2019; Raghavan et al., 2022).

## Conclusion

The journey to a placental malaria vaccine has been defined by the persistent challenge of VAR2CSA, a target that is as biologically essential as it is immunologically elusive. For decades, the field has been locked in an arms race with a parasite that utilizes extreme polymorphism and structural complexity to stay one step ahead of vaccine-induced immunity. However, as we have outlined in this review, the tools to break this stalemate are now in hand.

We stand at the precipice of a renaissance in maternal immunization. The convergence of high-throughput proteogenomics, structural biology, and next-generation delivery platforms offers a new roadmap. By expanding the antigenic atlas beyond VAR2CSA to include the cryptic understory of SURFINs, PHISTs, and other conserved effectors, we can dismantle the parasite’s sequestration machinery from multiple angles. When coupled with the precision of structure-guided epitope engineering and the breadth of mosaic nanoparticle or mRNA delivery, this multi-pronged strategy promises to replace the “leaky” protection of current candidates with a robust, pan-strain firewall.

The path forward will require a broader and more integrative strategy. It demands a holistic approach that integrates molecular parasitology with systems serology and innovative clinical trial design. If successful, the impact will extend far beyond the laboratory. A highly efficacious placental malaria vaccine would not only safeguard the lives of millions of primigravid women and their infants but also serve as a powerful tool for equity, ensuring that the most vulnerable populations are not left behind in the global malaria elimination agenda. The conceptual framework has been expanded; the next priority is to translate these insights into effective vaccine design and testing.

## Data Availability

No primary datasets were generated or analyzed during the current study. All data discussed in this review are obtained from publicly available, previously published sources cited in the article.
