# Supplementary material for: Beyond VAR2CSA: redefining the antigenic landscape of placental malaria vaccines
Source: Open Res Afr. 2026 Apr 30;9:18. [Version 1] doi: 10.12688/openresafrica.16523.1 (PMC13332378; doi:10.12688/openresafrica.16523.1)
Supplement: Peer review history

# Reviewer report: Reviewer response for version 1

**DOI:** 10.21956/openresafrica.17711.r34941

**Published:** 2026-07-03

**Authors:** Michael F. Ofori

**Affiliations:** 1 Noguchi Memorial Institute for Medical Research, College of Health Sciences,University of Ghana, Accra, Ghana

General Comments: This is a review article trying to highlight the importance  of developing vaccines for placental malaria with special emphasizes on other emerging vaccine candidate antigens other than VAR2CSA. It is a well written review paper and refreshing to read. The authors have done an extensive literature on antigenic landscape of placental malaria beyond VAR2CSA which has been the most extensive studied vaccine against placental malaria . They wanted to concentrate on other exported protein such as SURFINs, PHISTs, PfRON3, and *Pf*CSA-L, as critical targets to see if they could help in overcoming some of the challenges associated with the already in trial VAR2CSA vaccines , making the article very essential to the malaria research community.

 Again, the placental malaria situation was well described and appropriate references sited but I was not too sure about your accession that other vaccine candidates should also be looked at, as so much emphasis has been placed on the VAR2CSA vaccine candidates. In as much as I support your argument that those candidate vaccines should also be looked at, evidence backing them is not strong. There is data demonstrating that pregnant women who carry parasites until delivery do clear these parasites immediately after delivery indicates that the parasite infecting the pregnant woman is of placental focus. Probably if you change the language a bit to make it speculatory it will be helpful.

 How the review was done was not clearly described as it only appeared in the abstract- I suggest that you elaborate a little on the methodology – It will be important to demonstrate that the data used in arriving at these conclusions are not biased towards the papers selected for this review. Example, your data on antibody responses by pregnant women to SURFIN antigens had only 12 primigravidae women as compared to few multigravidae women- although the total samples used was 53 not all was used for the analysis.

 In your conclusion the suggestion of  inclusion of SURFIN is too strong because their function is still not very clear as you stated.

Is the review written in accessible language?

Yes

Are all factual statements correct and adequately supported by citations?

Yes

Are the conclusions drawn appropriate in the context of the current research literature?

Yes

Is the topic of the review discussed comprehensively in the context of the current literature?

Yes

Reviewer Expertise:

Immunology and Parasitology

I confirm that I have read this submission and believe that I have an appropriate level of expertise to confirm that it is of an acceptable scientific standard.

# Reviewer report: Reviewer response for version 1

**DOI:** 10.21956/openresafrica.17711.r34946

**Published:** 2026-06-24

**Authors:** Stephen Rogerson

**Affiliations:** 1 The University of Melbourne, Parkville, VIC, Australia

This article by Kanoi and colleagues reviews some characteristics of potential placental malaria vaccines, including both the established candidate VAR2CSA (and variants of it) and some other antigens which have varying levels of evidence regarding their importance in placental malaria.  A vaccine to prevent malaria and placental infection in pregnant women would undoubtedly be a major advance in malaria prevention. Beyond VAR2CSA the evidence for the potential of the other antigens discussed is far from established, and the article would benefit from a Table outlining important next steps for each, if they are to be pursued.

 Major comments

1. Somewhat surprisingly, while the authors do discuss the published PfSpz study of preconceptional protection, they neither report on the degree of protection against malaria in pregnancy it conferred, nor do they consider that vaccination of women of childbearing age could be a suitable target group for trials of RTS,S or R21 vaccination, although these products are at least licensed for use in young children.
2. The referencing in this paper is rather a mixed bag. While it is mostly appropriate, there are some places where the authors reference studies which do not themselves address the topic. Examples include MF Duffy et al 2016 (original research on antibody to PfEMP1 in non-pregnant people) and Keitani et al 2022 (which reports on PfCSA-L, but not that I can see on PFHISTb or PTEF).
3. Some of the candidate antigens identified are presented in Table 2. If they are important target of pregnancy-specific immunity it would be more valuable to report gravidity dependent recognition than seroprevalence on pregnancy overall- which tells us little. The case is not always made that they are really PMV candidates.
4. For the authors’ candidates, the paper would be strengthened by laying out what type of additional information would help make the case for these candidates. Are they pregnancy specific? Are they protective? If naturally acquired immunity is not thought to be a useful surrogate, how would one proceed to evaluate their possible role?
5. It is not clear why the authors include PAM1.4, a monoclonal antibody. While passive immunisation might have a place in pregnancy malaria prevention, the authors don’t set up this idea. Moreover PAM1.4 does not block binding to CSA and its other functional activities are not established, so it does not presently seem to be a strong candidate, while better monoclonals might be.
6. Page 12 first paragraph, rather than just citing Srinivasan, it would be better to cite some of the growing literature on both binding inhibition and opsonizing antibodies and their importance in naturally acquired immunity to PM/
7. Last paragraph, as highlighted elsewhere there are a few more steps before we decide which of the potential PMV candidates to take into vaccine studies.

Minor comments:

1. Section 1.1 should read “a mosquito bite”. Note not all pre-erythrocytic vaccine candidates are on sporozoites.
2. In section 1.1 state what the efficacy is again- infection? Malaria?
3. Page  5 para 1 – this is a very long opening sentence and should be split up and simplified. Some things are capitalised that shouldn’t be; capital missing for Duffy
4. Page 5 para 2: It is not a “PMV vaccine” – like it isn’t “an HIV virus”
5. Same paragraph – not clear what’s meant by “safe antibodies”. Again, shorter sentences would be clearer for the reader.
6. Top page 6 spell out abbreviation CFA/IFA
7. Sentence “additional challenges” needs revision so parts agree.
8. Page 11, second last paragraph, define ns EMPEM

Is the review written in accessible language?

Yes

Are all factual statements correct and adequately supported by citations?

Partly

Are the conclusions drawn appropriate in the context of the current research literature?

Partly

Is the topic of the review discussed comprehensively in the context of the current literature?

Yes

Reviewer Expertise:

Malaria pathogenesis and immunity, including antibody protection from placental malaria

I confirm that I have read this submission and believe that I have an appropriate level of expertise to confirm that it is of an acceptable scientific standard, however I have significant reservations, as outlined above.

# Reviewer report: Reviewer response for version 1

**DOI:** 10.21956/openresafrica.17711.r34517

**Published:** 2026-05-20

**Authors:** Hideyuki Iriko

**Affiliations:** 1 Kobe University, Kobe, Hyōgo, Japan

This review addresses an important and timely topic by exploring vaccine strategies for placental malaria (PM) beyond the traditional focus on VAR2CSA. The manuscript is well organized and presents a comprehensive overview of emerging antigen candidates, including SURFINs, PHIST proteins, PfRON3, and PfCSA-L, together with recent advances in proteogenomics, structural vaccinology, and modern vaccine delivery platforms. The inclusion of Table 2, which summarizes genomic, proteomic, functional, and immunological evidence for candidate antigens, is a particular strength and provides a useful framework for rational vaccine design. Overall, this is a thoughtful and forward-looking review that will be of interest to researchers in malaria immunology and vaccine development. I believe the manuscript is suitable for indexing after minor revisions.

 In the Background section, the authors state that the limitations of VAR2CSA have “hindered the development of a highly efficacious PM vaccine, necessitating a paradigm shift.” While antigenic diversity and structural complexity are genuine challenges, this wording may understate the progress achieved with VAR2CSA-based vaccine candidates such as PRIMVAC and PAMVAC, which have demonstrated acceptable safety profiles and immunogenicity in early clinical trials. I suggest moderating this statement to acknowledge these advances while emphasizing the rationale for investigating complementary vaccine targets.

 The Methods section states that the review examines the existing literature, but it does not specify whether this is a narrative review or whether a systematic search strategy was used. For transparency, the authors should clarify the type of review and, if appropriate, briefly describe the literature search strategy and inclusion criteria.

 The inclusion of Table 2 is a major strength of the manuscript. It effectively summarizes the available genomic, proteomic, functional, and immunological evidence for a broad range of candidate antigens. However, the strength of evidence varies substantially among the listed proteins. In particular, SURFINs, PHIST proteins, PfRON3, and PfCSA-L are intriguing candidates, but the current evidence supporting them as placental malaria vaccine targets remains preliminary compared with the extensive data available for VAR2CSA. In most cases, their direct roles in placental sequestration and their ability to induce functional adhesion-blocking antibodies have not yet been conclusively demonstrated. Accordingly, terms such as “critical targets” may overstate the current level of validation. More cautious wording, such as “promising candidate antigens” or “emerging targets,” would better reflect the evidence summarized in Table 2.

 The Conclusion provides an inspiring outlook for the field and effectively highlights the potential of emerging technologies. However, several statements are highly aspirational and may overstate the maturity of the current evidence base. For example, phrases such as: “we stand at the precipice of a renaissance,” “dismantle the parasite’s sequestration machinery from multiple angles,” and “a robust, pan-strain firewall” are rhetorically compelling but somewhat speculative, particularly given that many of the proposed non-VAR2CSA antigens remain at an early stage of validation. I suggest moderating the tone to more clearly distinguish between established findings and future possibilities. This would strengthen the scientific objectivity of the review while preserving its forward-looking perspective.

 Minor Comments

1. In the Methods section, “placental malaria ,and” should be corrected to “placental malaria, and.”
2. In the Results section, “This Review” should be changed to “This review.”
3. In Section 2.2, the term “vaccineworthy targets” is somewhat informal; “promising vaccine targets” or “candidate vaccine antigens” would be more appropriate.
4. In the Conclusions section, “placental malaria (PM)” does not need to be redefined.
5. The statement describing the inflammatory consequences of placental malaria is supported by well-established literature. However, Reference Mwai et al. (2024), published in Open Research Europe, currently has a mixed peer review status ("1 approved, 1 approved with reservations, 2 not approved"). I recommend replacing this citation with a more established peer-reviewed review or original articles.

Is the review written in accessible language?

Yes

Are all factual statements correct and adequately supported by citations?

Yes

Are the conclusions drawn appropriate in the context of the current research literature?

Yes

Is the topic of the review discussed comprehensively in the context of the current literature?

Yes

Reviewer Expertise:

parasitology

I confirm that I have read this submission and believe that I have an appropriate level of expertise to confirm that it is of an acceptable scientific standard, however I have significant reservations, as outlined above.
